# Unravelling the patterns of host immune responses in *Plasmodium vivax* malaria and dengue co-infection

**DOI:** 10.1186/s12936-015-0835-8

**Published:** 2015-08-14

**Authors:** Vitor R R Mendonça, Bruno B Andrade, Ligia C L Souza, Belisa M L Magalhães, Maria P G Mourão, Marcus V G Lacerda, Manoel Barral-Netto

**Affiliations:** Laboratório Integrado de Microbiogia e Imunoregulação (LIMI), Centro de Pesquisas Gonçalo Moniz, Fundação Oswaldo Cruz (FIOCRUZ), Salvador, Brazil; Faculdade de Medicina, Universidade Federal da Bahia, Salvador, Brazil; Fundação de Medicina Tropical Dr. Heitor Vieira Dourado, Manaus, Brazil; Universidade do Estado do Amazonas, Manaus, Brazil; Instituto de Investigação em Imunologia, Instituto Nacional de Ciência e Tecnologia, São Paulo, Brazil

**Keywords:** Immune response, Co-infection, Dengue, *Plasmodium vivax*, Malaria

## Abstract

**Background:**

Concurrent malaria and dengue infection is frequently diagnosed in endemic countries, but its immunopathology remains largely unknown. In the present study, a large panel of cytokines/chemokines and clinical laboratory markers were measured in patients with *Plasmodium vivax* and dengue co-infection as well as in individuals with malaria or dengue mono-infections in order to identify biosignatures of each clinical condition.

**Methods:**

Individuals from the Brazilian Amazon were recruited between 2009 and 2013 and classified in three groups: vivax malaria (n = 52), dengue (n = 30) and vivax malaria and dengue co-infection (n = 30). *P. vivax* malaria was diagnosed by thick blood smear and confirmed by PCR; dengue cases were detected by IgM ELISA or NS1 protein. The plasma levels of cytokines and chemokines were determined by multiplex assay.

**Results:**

Individuals with malaria and dengue co-infection displayed lower levels of platelets and haemoglobin than those with malaria or dengue mono-infections (p = 0.0047 and p = 0.0001, respectively). The group of individuals co-infected exhibited the highest median concentrations of IFN-γ, IL-6, CCL4 than the mono-infected groups. Network analyses of plasma cytokines/chemokines revealed that malaria and dengue co-infection exhibits a distinct immune profile with critical roles for TNF, IL-6 and IFN-γ. Further, parasitaemia levels displayed positive significant interactions with IL-6, CCL4 and IL-10 in the group of patients co-infected with malaria and dengue. No differences were observed in distribution of dengue virus serotypes and *Plasmodium* parasitaemia levels between the groups.

**Conclusions:**

The findings described here identify unique patterns of circulating immunological markers in cases of malaria and dengue co-infection and provide insights on the immunopathology of this co-morbid condition.

**Electronic supplementary material:**

The online version of this article (doi:10.1186/s12936-015-0835-8) contains supplementary material, which is available to authorized users.

## Background

Malaria and dengue fever are the most frequent arthropod-borne diseases in the world. Every year *Plasmodium* infection is responsible for around one million deaths, mainly in children [1]. It is estimated that two-fifths of the world population are at risk of dengue fever with 50–100 million cases each year worldwide [2, 3]. Both malaria and dengue fever exhibit dramatically similar geographic distribution (mostly in tropical and sub-tropical regions) and the detection of patients with concurrent malaria and dengue infections is not rare [4–19].

Previous studies have reported a frequent presence of malaria and dengue co-infections in different countries and implied that this fact creates challenges for reliable clinical diagnosis due to the overlap of major symptoms with malaria or dengue mono-infections [4, 6–8, 11, 12, 14, 16]. Recently, observations from a case series of patients with dual malaria and dengue infections performed at the Brazilian Amazon indicated that co-infection can potentially result in a more severe disease presentation [10]. The status of host immune activation profile in patients with dengue and malaria co-infection, which may explain the clinical features of this condition, has not been systematically investigated.

The immunopathogeneses of dengue and malaria display common features, which include the production of multiple cytokines and the balance between pro-inflammatory and anti-inflammatory responses may regulate the clinical spectrum of these infections [20–25]. Importantly, circulating cytokines as well as other inflammatory mediators may be used as biomarkers for an early diagnosis or for prediction of unfavourable clinical deterioration and poor prognosis or treatment responses [26]. Moreover, understanding the key factors associated with increased morbidity may lead to development of host-directed therapy focused on the modulation of pathological immune responses and better clinical prognosis. The present study performs for the first time a detailed exploratory description of the systemic immune profile of individuals presenting with *Plasmodium vivax* malaria and dengue co-infection as well as in subjects with *P. vivax* or dengue mono-infections.

## Methods

### Study design and participants

Outpatients with an acute febrile syndrome who sought care in a reference hospital (Fundação de Medicina Tropical Doutor Heitor Vieira Dourado, FMT-HVD) in Manaus, in the Brazilian Amazon, were recruited between 2009 and 2013. Malaria individuals were diagnosed by blood thick smear and those with *P. vivax* confirmed by PCR were recruited. Dengue subjects were diagnosed by: NS1 and RT PCR (Kit Platelia™ Dengue NS1 Ag, Bio-Rad, France) in individuals with fewer than 6 days of fever, or by the detection of IgM ELISA as descrided by Kuno et al. [27] in individuals with more than 7 days of fever. All dengue-positive individuals were recruited and had the identification of virus serotype by RT PCR. Co-infected subjects with *P. vivax* malaria and dengue were also recruited. All patients with microscopic or molecular diagnosis of malaria caused by *Plasmodium falciparum* or *P. vivax* and *P. falciparum* co-infection (mixed infection), patients with serologic diagnosis of viral hepatitis (A, B, C, and D), HIV, and leptospirosis were excluded. Patients with complications of dengue or malaria according to WHO criteria [28] were excluded from this study. All malaria cases were treated following the guidelines of the National Foundation of Health, Brazil, with chloroquine for three days and primaquine (0.5 mg/kg/day) for 7 days. Dengue patients were treated according to their symptomatology. No individuals were treated for malaria or/and dengue 30 days before the blood collection and participation in this study.

### Ethics statement

All clinical investigations were conducted according to the principles expressed in the Declaration of Helsinki. Written informed consent was obtained from all participants before enrolling into the study. This study was approved by the Ethics Committee of the FMT-HVD (protocol numbers: 2009/15243 and 39163/2012).

### Plasma measurements

Blood was obtained by venopuncture at the study enrolment and heparinized plasma was separated by centrifugation and stored at −70 °C until use in immunoassays. Circulating levels of several cytokines and chemokines, including IL-1β, IL-2, IL-4, IL-5, IL-6, IL-7, IL-8, IL-10, IL-12p70, IL-13, IL-17, IFN-γ, TNF, CCL2, CCL4, GCSF, GMCSF, were measured using a single multiplex assay according to the manufacturer’s protocol (BIO-RAD, Hercules, CA, USA). The clinical laboratory markers haemoglobin (HB), haematocrit (HT), platelets (PTL), aspartate amino-transferase (AST) and alanine amino-transferase (ALT) were measured in fresh plasma/serum samples at the Clinical Laboratory facility from the FMT-HVD (Manaus, Brazil).

### Network analysis

The inferential networks were generated from Spearman correlation matrices containing values of each biomarker measured in the plasma samples. The values were input in JMP 10.0 software (SAS, Cary, NC, USA). Each mediator is selected as a target, and the software performs a search within the other mediators for those that are correlated, with the target calculating a correlation matrix using Spearman rank tests. As a result, the features related to the selected target are linked. The links shown in the networks represent statistically significant Spearman rank correlations (P < 0.05). In order to analyse the structure of the biomarker networks, the network density was calculated, which is, in the context of this study, the ratio of the number of edges inferred in the network over the total number of possible edges between all pairs of nodes [24]. The density measure is defined as follows: density = L/(N (N − 1)/2), in which L is the number of observed edges (i.e., Spearman correlations with P < 0.05) and N is the total number of the nodes in the network. The density is normalized, ranging between 0 (no edges in the network) and 1 (all possible edges present). The networks figures were customized using the Ingenuity Systems Pathway Analysis software (Ingenuity Systems, Redwood City, CA, USA) and Adobe Illustrator (Adobe Systems Inc.).

### Data analysis

In the exploratory analysis of the data, frequency tables were constructed and the Chi square test was applied to evaluate the association between categorical variables. The continuous variables were tested for Gaussian distribution within the total sample using D’Agostino and Pearson omnibus normality test. All variables were not under normal distribution, and non-parametric tests were used instead. In this context, Kruskal–Wallis with Dunn’s multiple comparison (when three groups were compared) or Mann–Whitney tests (when two groups were compared) were used to assess the differences between the clinical groups. Multinomial regression analyses adjusted for age and gender were performed to test associations between the laboratory measures (below or above the median values of the entire study population) and the different clinical conditions evaluated (malaria, dengue or co-infection). A hierarchical cluster analysis using the Ward’s method was performed to test if a combination of different immune-related biomarkers could cluster the study groups separately. The statistical analyses were performed using the programs GraphPad Prism 6.0 (GraphPad Software Inc., USA), SPSS 19.0 (IBM, Armonk, NY, USA) and JMP 11.0 (SAS, Cary, NC, USA). A *p* value lower than 0.05 was considered statistically significant.

## Results

### Characteristics of the study participants

After clinical and microbiological assessments, individuals were grouped as vivax malaria (n = 52), dengue (n = 30) and co-infected vivax malaria/dengue (n = 30). Most participants from the malaria group were male (80.77 %, n = 42) while the groups of co-infected and dengue patients exhibited a predominance of females (70.00 %, n = 9 for both; p < 0.0001). There were no differences with regard to age between the groups (p = 0.0724; Table 1) and also no statistically significant discrepancy in parasitaemia levels between the groups of individuals infected with *P. vivax* (p = 0.4912; Table 1). Moreover, the majority of individuals infected with dengue had DENV2 serotype with no differences in distribution of virus serotypes in the groups of individuals with dengue or dengue and malaria co-infection (Table 1).

**Table 1:** Demographic characteristics and laboratory measures of the participants

	Malaria	Co-infection Mal/Deng	Dengue	P value		
	(n = 52)	(n = 30)	(n = 30)	All groups	Malaria vs. co-infection	Dengue vs. co-infection
Male-no. (%)	42 (80.77)	09 (30.00)	09 (30.00)	<0.0001^a^	<0.0001^a^	1.000^a^
Median (IQR) age (year)	36.00 (26.25–43.75)	31.11 (20.80–44.74)	42.50 (30.00–52.25)	0.0724^b^	0.4471^b^	0.0574^b^
Median (IQR) of parasitaemia (parasites/uL)	3,022 (985.2–9,313)	4,262 (1,595–12,199)	–	–	0.4912^b^	–
Dengue serotypes-no. (%)						
DENV1		3 (10.00)	1 (3.33)	–	–	0.2247^a^
DENV2		18 (60.00)	21 (70.00)			
DENV3		1 (3.33)	4 (13.33)			
DENV4		8 (26.67)	4 (13.33)			
Median of laboratory measures (IQR)						
Haemoglobin (g/dL)	13.20 (12.50–14.20)	12.95 (11.90–14.45)	15.00 (13.40–15.95)	0.0047^b^	0.4819^b^	0.0038^b^
Haematocrit (%)	43.35 (40.43–45.98)	42.05 (37.80–45.98)	43.90 (40.65–46.90)	0.5803^b^	0.3732^b^	0.3817^b^
Platelets (by mm^3^)	102,000 (65,000–131,500)	87,500 (59,000–114,250)	186,500 (124,000–229,750)	0.0001^b^	0.1079^b^	<0.0001^b^
AST (IU/L)	67.50 (50.00–91.00)	47.00 (31.50–72.50)	47.00 (28.00–147.00)	0.0186^b^	0.0048^b^	0.9315^b^
ALT (IU/L)	33.00 (20.00–49.75)	69.00 (46.00–95.50)	67.00 (29.50–119.00)	<0.0001^b^	< 0.0001^b^	0.9043^b^

*IQR* interquantile range.

^a^Categorized variables were compared using Chi square test or Fisher exact test.

^b^Continuous variables were compared using Mann–Whitney for two groups or Kruskal–Wallis test with Dunn’s multiple comparison test for three groups or more.

### Differential expression of clinical laboratory parameters reveals unique patterns of associations with infection status

**Fig. 1:**
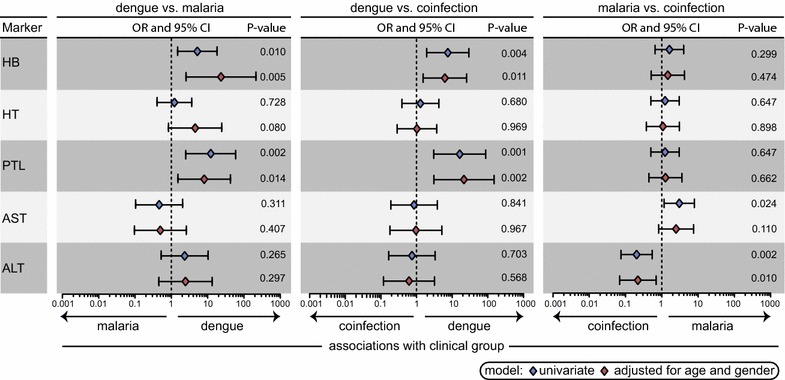
Discrimation of malaria, dengue and co-infection groups by laboratory measures. Differentiation between dengue *vs* malaria, dengue vs co-infection and malaria vs co-infection groups were done by laboratory measures—*HB* haemoglobin, *HT* haematocrit, *PTL* platelets, *AST* aspartate aminotransferase, *ALT* alanine aminotransferase—through multinominal regression analysis with calculation of odds ratios (OR) and 95 % confidence intervals (CI), represented by the *icons* and *bars*, respectively (**a**). *Red icons* represent OR adjusted for age and gender and *blue icons* were unadjusted (univariate) (**a**).

Univariate analyses revealed that individuals co-infected with malaria and dengue exhibited lower levels of HB and PTL than those with malaria or dengue mono-infections (p = 0.0047 and p = 0.0001, respectively; Table 1). On the other hand, plasma AST levels were elevated whereas ALT concentrations were decreased in individuals with malaria mono-infection compared to those with dengue or co-infection (p = 0.0186 and p < 0.0001, respectively; Table 1). Multinominal regression analyses adjusted for age and gender uncovered that higher levels of HB (adjusted OR: 23.344 95 % CI 2.534–215.023, p = 0.005) and PTL (adjusted OR: 8.065 95 % CI: 1.527–42.612, p = 0.014) were associated with dengue when compared to malaria (Fig. 1). Furthermore, higher levels of HB (adjusted OR: 6.264 95 % CI 1.535–25.553, p = 0.011) and PTL (adjusted OR: 21.471 95 % CI 3.077–149.827, p = 0.002) were associated with dengue mono-infection compared with dengue and malaria co-infection (Fig. 1). Higher levels of AST (unadjusted OR: 3.030 95 % CI 1.160–7.914, p = 0.024) and low levels of ALT (adjusted OR: 0.219 95 % CI 0.069–0.695, p = 0.010) were associated with malaria mono-infection compared with the malaria/dengue co-infection condition (Fig. 1).

### Networking the immune response

**Fig. 2:**
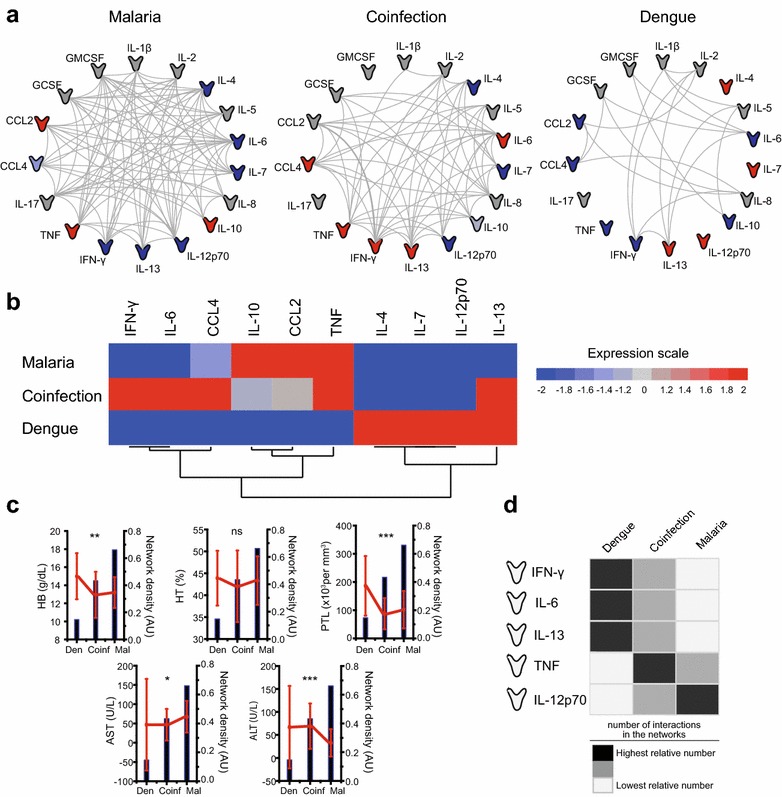
Networks of candidate immune-related biomarkers during malaria, dengue or co-infection. Plasma levels of several immune-related (cytokines, chemokines) biomarkers were measured in malaria, dengue and co-infection subjects. Each connecting line represents a significant interaction (P < 0.05) detected by Spearman’s correlation test (**a**). All interactions had positive correlations. A heat map was designed to depict the overall pattern of expression of immune markers in the different outcomes by the median value of each parameter (**b**). A two-way hierarchical cluster analysis (Ward’s method) of immune molecules by clinical group was performed (**b**). Biomarkers that had the same median in the three groups were excluded from the heat map and cluster analysis. The *colours* shown for each *symbol* represent the fold variation from the median values calculated for each marker (**a**, **b**). The distribution of haemoglobin (HB), haematocrit (HT), platelets (PTL), aspartate aminotransferase (AST), alanine aminotransferase (ALT) in different clinical groups is shown in red symbols (medians and interquartile ranges) whereas the values for network densities are shown as *black bars* (**c**). The variation of HB, HT, PTL, AST, and ALT according to the groups was assessed using the Kruskal–Wallis test (***P < 0.001; **P < 0.01; *P < 0.05; ns = non-significant) (**c**). The five immune-related biomarkers with the highest number of interactions in all three groups were chosen (IFN-γ, IL-6, IL-13, TNF, and IL-12) and the relative number of interactions of these biomarkers was calculated according to each group (**d**). *Dark grey rectangles* represent the highest relative number of connections, *light grey* *rectangles* the medium relative number and *white rectangles* the lowest relative number of hits between molecules (**d**).

A panel of 17 cytokines and chemokines was used to build networks demonstrating the interactions between the candidate biomarkers in each group (Fig. 2a). The distributions of plasma concentrations of each cytokine or chemokine amongst the different clinical groups are provided (see Additional file 1). The network analysis revealed an absence of negative correlations between the candidate biomarkers in each one of the clinical groups and only statistically significant positive correlations were detected (Fig. 2a). Strikingly, the densities of the networks from each clinical group were dramatically different (Fig. 2a). The group of malaria mono-infection exhibited highest density of interactions (network density: 0.661) followed by the groups of co-infected patients (network density: 0.4338) and dengue mono-infection (network density: 0.147) (Fig. 2a). P values and Spearman rank values for each correlation between the immune biomarkers according to study groups are detailed (see Additional file 2). Moreover, the simultaneous assessment of several immune-related markers revealed relative differences in plasma concentrations that resulted in unique biosignatures, which could highlight differences between the study groups in an hierarchical cluster analysis (Fig. 2b). Amongst the clinical groups evaluated, the group of individuals with malaria and dengue co-infection exhibited the highest median concentrations of IFN-γ, IL-6, CCL4 (Fig. 2b). The group of malaria mono-infected patients exhibited a biosignature composed by higher levels of IL-10 and CCL2 whereas the group of dengue mono-infected individuals displayed a signature with high expression of IL-4, IL-7 and Il-12 in plasma (Fig. 2b). Furthermore, TNF was found elevated in both groups of malaria mono-infection and co-infection with dengue whereas IL-13 was detected in higher amounts in the groups of dengue mono-infection and co-infection (Fig. 2b). While investigating the relationships between changes in clinical laboratory markers and the inflammatory environment assessed by network densities, it was observed that HB, PTL and ALT displayed a general trend to decrease in concentration values according to the increase of network’s complexities (Fig. 2c). Nevertheless, AST levels tended to increase following the density of correlations between the markers in the groups (Fig. 2c). No significant difference was observed in variations of HT levels and its associations with network densities (Fig. 2c).

Amongst the 17 immune-related biomarkers assessed in the network analyses, five cytokines exhibited the highest number of interactions (statistically significant Spearman correlations) when all the study groups were considered together: IFN-γ (participated in 7.40 % of all interactions), IL-6 (8.88 % of all interactions), IL-13 (7.10 % of all interactions), TNF (6.50 % of all interactions) and IL-12p70 (6.50 % of all interactions) (Fig. 2d). In order to assess if the number of network connections involving each one of these five cytokines could highlight differences between the clinical groups, the percentage of edges involving with each molecule related to the overall number of edges in the network was calculated. Interestingly, IFN-γ, IL-6 and IL-13 displayed the highest relative number of network interactions in the group of dengue mono-infected patients, whereas IL-12p70 exhibited the highest relative number of interactions in the malaria mono-infection group (Fig. 2d). TNF exhibited the highest number of interactions in the group of patients with malaria and dengue co-infection (Fig. 2d). These results argue that unique immune signatures involving plasma cytokine levels are able to highlight differences that distinguish malaria, dengue or dual malaria and dengue infection.

**Fig. 3:**
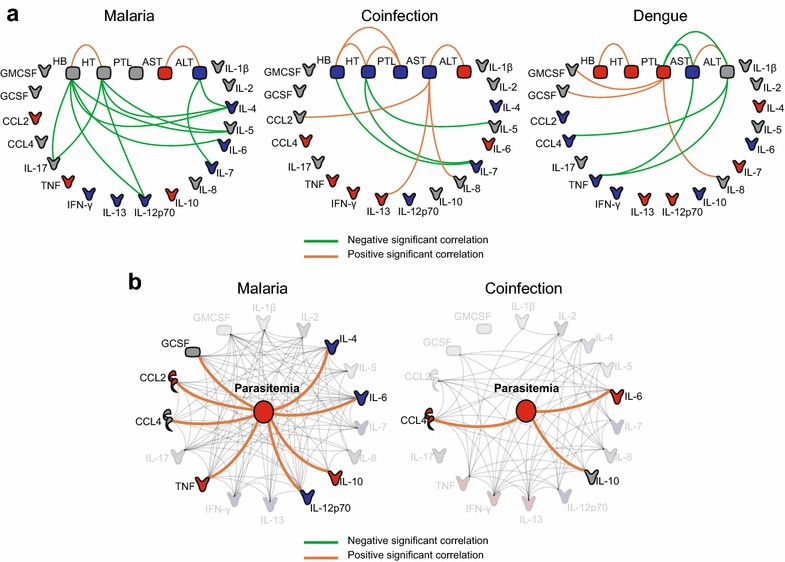
Associations between laboratory parameters, parasitaemia and immune-related biomarkers. Statistically significant correlations between laboratory markers (**a**) or parasitaemia (**b**) and immune-related biomarkers are shown for the different groups. The correlations were assessed using the Spearman rank test. The interactions involving parasitaemia shown in (**c**) are plotted on top of the host interactome. *Green lines* represent negative significant (P < 0.05) correlations and *orange lines*, positive significant correlations.

The next step was to uncover the interactions between clinical laboratory markers and the immune-related molecules. In all the study groups, HB exhibited positive associations with HT whereas ALT exhibited positive correlations with AST (Fig. 3a). It was found that HB and HT displayed several negative significant interactions mainly with IL-4, IL-5, IL-12p70, and IL-17, whereas ALT interacted negatively with IL-4 and IL-7 in the malaria group (Fig. 3a). In the group of patients with malaria and dengue co-infection, HB and HT displayed negative associations with IL-7, whereas AST exhibited positive interactions with CCL2, IL-13 and IL-8 (Fig. 3a). Noteworthy, it was observed that only in the dengue mono-infection group did PTL display interactions with immune markers (positive interactions with GMCSF, GCSF and IL-8), suggesting a major role for this molecule in this group (Fig. 3a). Furthermore, AST and ALT displayed negative associations with TNF in the network of the dengue mono-infection group (Fig. 3a). In the *P. vivax*-infected groups, the associations between parasitaemia and the immune markers were also studied (Fig. 3b). It was observed that *P. vivax* parasitaemia displayed positive significant interactions with IL-6, CCL4 and IL-10 in the group of patients co-infected with malaria and dengue, while this parameter exhibited several positive correlations with many immune markers (GCSF, CCL2, CCL4, TNF, IL-12p70, IL-10, IL-6, and IL-4) in the group of malaria mono-infected subjects, suggesting a major role for parasitaemia in the immune profile in this clinical condition (Fig. 3b). P values and Spearman rank values for each correlation between the immune biomarkers and laboratory measures or parasitaemia are provided (see Additional file 3).

## Discussion

Malaria or dengue immunology has been studied extensively in a diverse range of scenarios, however, the profile of the immune responses in individuals co-infected with *Plasmodium* and dengue virus has not been systematically explored. In the present study, a large panel of cytokines and chemokines as well as several clinical laboratory markers used to assess degree of disease severity and inflammation-driven tissue damage have been investigated in plasma samples of patients with malaria, dengue or co-infection in order to identify immune signatures associated with each infection status. Network analyses revealed that cases of malaria and dengue co-infection exhibit a unique immune profile with a special role for TNF, IL-6, IFN-γ, and IL-7. In addition, the analysis herein further revealed a signature profile in which *P. vivax* parasitaemia levels display positive significant interactions with IL-6, CCL4 and IL-10 in patients with this co-infection, which was not observed in malaria mono-infected individuals.

Some studies have studied laboratory measurements in subjects with dual malaria and dengue infections [4, 6, 11]. HB and PTL are usually shown to be decreased in individuals co-infected with malaria and dengue and AST has been shown to be higher in individuals with malaria compared with co-infected cases [4, 6, 11]. These findings are similar to the results described in the present study. When dengue was compared to malaria mono-infection or co-infected cases, the former had higher values of HB and PTL. Malaria infection is frequently associated with anaemia as its causative parasite has a blood stage and causes intense intravascular haemolysis. One major complication associated with both dengue and malaria disease is thrombocytopaenia and the findings from the present study indicate that *P. vivax* infection may cause more severe thrombocytopaenia (with lowest values in co-infected cases) compared to dengue fever mono-infection [29–31]. Other studies have also reported that platelet counts were lower in malaria patients than dengue patients [15, 32]. On the other hand, laboratory markers assessed in the current study showed similar values between malaria mono-infection and cases of malaria and dengue co-infection, with a major difference in ALT levels (and in less extension to AST), which were more elevated in the patients with co-infection, and no significant difference was observed in parasitaemia levels. The relationship of AST and ALT and parasitaemia with clinical manifestations of mono- or co-infection of malaria and dengue is not well established. A previous study showed higher levels of AST and ALT and parasitaemia in malaria mono-infection compared to malaria and dengue co-infection [11]. Nevertheless, another report showed no differences in concentrations of AST and ALT as well as levels of parasitaemia between malaria mono-infected patients and those with malaria and dengue co-infection [6]. Differences in patient populations and parasite strains could explain these discrepancies. Parasitaemia levels may have an important role in predicting hepatic damage and need to be considered in malaria-infected subjects [25].

Heightened levels of TNF and IFN-γ have been systematically associated with increased clinical disease severity in malaria or dengue fever in many case series [20, 21, 24, 25, 33–35]. An important role for TNF and IFN-γ in the onset of malaria symptomatology as well as in pathological processes associated with platelet consumption, endothelial cell activation and haemorrhagic manifestations during dengue fever have been described previously [33, 36]. In the present study, increased TNF levels observed in the group of patients with malaria and dengue co-infection compared to malaria or dengue mono-infections, together with the significantly higher number of interactions in the cytokine/chemokine networks, argue that this cytokine may play a critical role in the pathogenesis of malaria and dengue fever comorbid condition. Noteworthy, *TNF* polymorphisms are common and may play a role in TNF levels in the context of malaria [37]. Moreover, amongst the clinical groups assessed herein, cases of malaria and dengue co-infection also exhibited the highest values of IFN-γ and IL-6. IL-6 has been implicated in the pathogenesis of severe cases of dengue as this cytokine enhances the production of anti-platelet or anti-endothelial cell auto-antibodies, as well as the induction of tissue plasminogen activator, leading to increased risk for bleeding [38, 39]. These findings on immune markers support the idea that co-infected cases may present with a more severe inflammation milieu and disease status compared to malaria or dengue mono-infections. Recently, it has been described that individuals with concurrent dengue fever and malaria from the Brazilian Amazon and French Guiana exhibited more severe disease clinical presentation than mono-infections [6, 9, 10].

In the present study, plasma levels of IL-4, IL-7, IL-12p70, and IL-13 were more elevated in subjects purely with dengue fever than in those with malaria mono-infection or malaria and dengue co-infection. Noteworthy, IL-12p70 has been previously associated with severity and protection during malaria [40–43], also polymorphism in this gene can influence this cytokine production during malaria [44]. IL-12p70 had the highest number of interacions in the malaria group and seems to influence the inflammatory milieu in this group. In addition, IL-13 had the highest relative number of interactions in the cytokine/chemokine network of the group of dengue mono-infection. IL-4 and IL-13 plasma levels were also previously found to be higher in dengue fever compared to malaria cases [23]. The increase in plasma expression of Th2 cytokines (i.e., IL-4, IL-13) may be associated with augmentation of vascular permeability and vascular leakage as seen in dengue haemorrhagic fever [45, 46]. Regarding malaria mono-infection, IL-10 and CCL2 were the immune markers with more relevant elevations in plasma. Maneekan et al. have described that IL-10 was statistically higher in malaria patients than in those with dengue fever [23]. Furthermore, IFN-γ/IL-10 ratio has been reported to increase proportionally to malaria clinical severity [25], and it is possible that cases of malaria and dengue co-infection presenting with lower levels of IL-10 and higher levels of IFN-γ may be reflected in an increased disease severity. Further studies, including a broader clinical spectrum of the infections explored here, are necessary to address this question. In a study about the immune profile of dengue fever and parvovirosis, high levels of CCL2 tended to be associated with parvovirus B19 infection as the same way as observed herein with malaria infection [47]. Therefore, different infectious agents disease may stimulate specific features of the host immune responses, which may result in unique immune signatures.

Although the group of individuals co-infected with malaria and dengue displayed an intermediate density of the immune network compared to the other clinical groups, this group displayed the lowest values of HB and PTL, thus indicating that the relationships between the inflammatory markers may not be influenced directly by the levels of these clinical laboratory markers. However, a potential role for HB and HT in regulation of the inflammatory environment in malaria and dengue comorbidity could not be entirely discarded, and the network findings indicate that these parameters were negatively correlated with IL-7 in this group. It has been reported that IL-7 plays an important role in CD4+ T cell immune responses against the dengue virus and this cytokine is also useful for maintaining the growth and antigen-specific cytotoxic activity of CD4+ human cytotoxic T lymphocyte clones [48]. In addition, AST seemed to be highly influenced by immune molecules in the malaria and dengue co-infected patients as this enzyme was positively correlated with CCL2, IL-13 and IL-8.

In the context of laboratory and immune markers correlations, the group of individuals presenting with malaria mono-infection displayed significant negative correlations between ALT and IL-4 and IL-7. Liver enzymes are highly inducible by the activation of immune responses and ALT was also correlated with TNF and superoxide dismutase-1 (SOD-1) levels in another case series of vivax malaria [49]. Furthermore, HB and HT had several negative interactions with inflammatory mediators (IL-17, IL-12, IL-5, IL-4) in the malaria group. These interactions may be explained as HB and HT levels as well as the immune response are directly influenced by intravascular haemolysis of red blood cell rupture upon *Plasmodium sp.* replication. Noteworthy, PTL positively interacted with immune markers (GCSF, GMCSF and IL-8) only in the group of patients with dengue mono-infection. It has been reported that exposure of monocytes from healthy volunteers to platelets from patients with dengue induced the secretion of IL-8 [50]. Thrombocytopaenia is often described in dengue fever (about of 23.6 % of cases) and it may play a role along with immune mediators in dengue immunopathology [51].

Parasitaemia was not different between the groups of individuals with malaria mono-infection and malaria and dengue co-infection, similarly to previous studies [6]. However, the network analysis uncovered that parasitaemia displayed more statistically significant interactions with cytokines/chemokines in the malaria mono-infection than in the group of co-infected patients. The immune response of individuals with concurrent dengue and malaria infections is also influenced by dengue viruses and this may explain the lower number of interactions of parasitaemia in this group. Moreover, one limitation of the present report was that some co-infected individuals could have had undiagnosed asymptomatic malaria, which can sometimes be diagnosed only with molecular methods. It is well described that asymptomatic malaria individuals control parasitaemia to very low levels (sometimes only detectable by molecular techniques) and this clinical immunity results in a reduced intensity of the inflammatory response when compared to symptomatic malaria cases [24, 25].

## Conclusion

Altogether, the findings of the present study indicate that concurrent malaria and dengue infections may cause a more severe disease compared to mono-infections as observed by laboratory and immune markers profiles. Overall, malaria and dengue co-infection displayed lower levels of platelets and haemoglobin and a specific immune signature with a special role for TNF compared to dengue or malaria. This detailed description of the immune response profile in subjects with malaria and dengue fever co-infection and the results depicted herein shed light into the immunopathology of this comorbid condition. Further prospective studies with larger samples and experimental models would be necessary to investigate the key mechanisms resulting in the biosignatures identified here in patients with malaria and dengue co-infection.

## Additional files

**Additional file 1.** Distribution of cytokines and chemokines according to malaria, co-infection and dengue groups.
**Additional file 2.** Correlation parameters between the immune biomarkers according to study groups.
**Additional file 3.** Correlation parameters between the immune biomarkers and laboratory measures or parasitaemia in the study groups.

## Abbreviations

FMT-HVD: Fundação de Medicina Tropical Dr Heitor Vieira Dourado
PCR: polymerase chain reaction
NS1: non-structural protein 1
RT PCR: real-time polymerase chain reaction
IgM: immunoglobulin M
ELISA: enzyme-linked immunosorbent assay
HIV: human immunodeficiency virus
WHO: World Health Organization
IL-: interleukin
IFN: interferon
TNF: tumour necrosis factor
CCL2: chemokine C–C motif ligand 2
CCL4: chemokine C–C motif ligand 4
GCSF: granulocyte colony stimulating factor
GMCSF: granulocyte macrophage colony stimulating factor
HB: haemoglobin
HT: haematocrit
PTL: platelets
AST: aspartate transaminase
ALT: alanine transaminase
DENV: dengue virus
OR: odds ratio
CI: confidence interval
CD: cluster of differentiation
SOD-1: superoxide dismutase 1
